# Relationship Between Hip and Groin Pain and Hip Range of Motion in Amateur Soccer and Australian Rules Football Players

**DOI:** 10.1177/23259671241277662

**Published:** 2024-10-25

**Authors:** Andrea B. Mosler, Joshua J. Heerey, Joanne L. Kemp, Adam I. Semciw, Matthew G. King, Rintje Agricola, Peter R. Lawrenson, Mark J. Scholes, Benjamin F. Mentiplay, Kay M. Crossley

**Affiliations:** † La Trobe Sport and Exercise Medicine Research Centre, La Trobe University, Melbourne, Australia; ‡ Nutrition and Health Innovation Research Institute, Edith Cowan University, Joondalup, Western Australia, Australia; § Discipline of Physiotherapy, Podiatry, and Prosthetics and Orthotics, La Trobe University, Melbourne, Victoria, Australia; ‖ Department of Orthopaedics and Sports Medicine, Erasmus University Medical Centre, Rotterdam, the Netherlands; ¶ School of Allied Health, University of Queensland, Brisbane, Australia; # Community and Oral Health Innovation and Research Centre, Metro North Health, Queensland, Australia; ** Discipline of Sport and Exercise Science, La Trobe University, Melbourne, Victoria, Australia; Investigation performed at La Trobe University, Melbourne, Australia

**Keywords:** sport, soccer, Australian Rules football, femoroacetabular impingement, adductor, rehabilitation

## Abstract

**Background:**

The relationship between hip/groin pain and hip range of motion (ROM) is unclear.

**Purpose:**

To explore the relationship between hip/groin pain and hip joint ROM and examine the influence of sex and cam morphology on this relationship.

**Study Design:**

Cross-sectional study; Level of evidence, 3.

**Methods:**

Included were 184 amateur soccer and Australian Rules football players (276 hips; 20% women; median age, 26 years; interquartile range, 24-30 years) with hip/groin pain >6 months and a positive flexion-adduction-internal-rotation (FADIR) test, and 50 matched asymptomatic control players (98 hips; 28% women, median age, 26 years; interquartile range, 23-31 years). Hip ROM measures were flexion, internal and external rotation at 90° of hip flexion, total rotation (internal and external), and bent-knee fall out (BKFO). Cam morphology was determined from anteroposterior pelvis or 45° Dunn radiographs, defined by an alpha angle ≥60°. Linear regression models with generalized estimating equations were used to examine the relationship between group (symptomatic and asymptomatic) and each ROM measure. Interaction terms (group × cam morphology or group × sex) were included to examine if relationships between group and hip ROM were influenced by cam morphology or sex. Where appropriate, models were adjusted for sex, age, and cam morphology.

**Results:**

An interaction between the relationship between group × cam and internal rotation ROM was found. Symptomatic players with cam morphology had lower internal rotation ROM than controls with cam morphology (adjusted mean difference [AMD] = −4.5°; 95% CI, −7.4° to −1.6°). Hip/groin pain was not associated with internal rotation ROM if cam morphology was absent. A significant interaction was also found for group × sex and BKFO and total rotation ROM. Symptomatic women had lower total rotation ROM than control women (AMD = −8.2°; 95% CI, −14.1° to −2.2°), but no difference was seen in men. BKFO range was lower in men with hip/groin pain compared with control men (AMD = 1.6 cm; 95% CI, 0.3-3.0 cm), but no difference was seen in women. Flexion and external rotation ROM did not differ between symptomatic and control hips.

**Conclusion:**

Cam morphology was an effect modifier of the relationship between hip/groin pain and internal rotation ROM. Sex-related differences were also observed in the relationship between hip/groin pain and hip ROM.

Range of motion (ROM) measurement is considered important in the clinical assessment of hip/groin pain.^7^ Measuring ROM can identify impairments, assist diagnosis, and record the effectiveness of interventions for people experiencing hip/groin pain.^29,37^ However, there is conflicting evidence on whether ROM differs between individuals with and without hip/groin pain.^6,8,27,29^ Therefore, the true clinical value of including ROM in the clinical assessment is currently unclear.^29^

Femoroacetabular impingement (FAI) syndrome is defined as a motion-related clinical hip disorder with a triad of hip/groin pain, clinical signs, and imaging findings of cam and/or pincer morphology.^10^ Restricted hip ROM, particularly internal rotation, is a key clinical sign associated with the diagnosis of FAI syndrome.^4,6,10,17,32,34^ It was theorized that the restricted hip ROM seen in people with FAI syndrome results from the abutment of the femoral “bump” formed by the cam morphology against the acetabulum.^4,9,24^ In 426 asymptomatic professional male soccer players, the presence of cam and/or pincer morphology was associated with less hip internal rotation ROM and range of bent-knee fall out (BKFO; a combined motion of hip flexion to 60°, abduction, and external rotation), supporting the association between bony hip morphology and hip ROM.^26^ Conversely, the presence of pain, regardless of bony morphology, was associated with reduced hip ROM.^36^ Therefore, it is unclear whether the bony morphology associated with FAI syndrome is associated with reduced ROM.^16^

Systematic review evidence described no relationship between hip ROM and symptoms in people with FAI syndrome.^8^ These findings were extended in 2 recent studies showing little difference in ROM between those with and without FAI syndrome^21^ or cam morphology without symptoms.^36^ Consequently, international clinical research experts at the International Hip Pain Research Network consensus meeting were unable to make clear clinical or research recommendations regarding the value or methods of assessing ROM in hip-related pain populations.^29^ While sex-specific analyses of hip ROM have rarely been published, preliminary evidence suggests that women athletes have greater hip ROM than men.^14^ Sex-related differences in biomechanics are evident in people with FAI syndrome,^19,20^ suggesting that sex should be considered when examining the relationship between hip/groin pain and ROM.

The primary aim of our study was to examine the relationship between hip/groin pain and hip joint ROM, accounting for cam morphology and sex. We hypothesized that symptomatic amateur soccer and Australian Rules football players would have less hip ROM than controls and that cam morphology would modify the relationship between group and ROM. We also hypothesized that there would be sex-related differences in the relationship between hip/groin pain and ROM.

## Methods

The Strengthening the Reporting of Observational Studies in Epidemiology (STROBE) guidelines were followed in the reporting of this study.^39^ Ethical approval was obtained from our institutions, and all included patients provided written informed consent prior to participating.

### Study Design and Participants

Participants with hip/groin pain were recruited as part of the Femoroacetabular impingement and hip OsteoaRthritis Cohort (FORCe) study.^5,11^ This ongoing prospective cohort study is investigating the relationship between hip/groin pain and changes in symptoms and hip joint structure on magnetic resonance imaging in amateur soccer and Australian Rules football players.^5^ Players included in the FORCe study participated in ≥2 sessions (training or competition) per week.^5,12^ The data collected at the baseline examination were used for this study. Players with self-reported hip and/or groin pain and a positive flexion-adduction-internal rotation (FADIR) test (symptomatic group) were recruited for this study between August 2015 and October 2018. These criteria were used to increase the likelihood that the study included participants with FAI syndrome. A group of 50 pain-free amateur players (also soccer and Australian Rules football) with a negative FADIR test were recruited as a control group.^11^

Eligibility criteria for symptomatic participants in this study have been described in detail previously and are included in Appendix Table A1.^5,11^ Briefly, players were classified as symptomatic if they met the following criteria in at least 1 hip: (1) reported hip (anterior, lateral, or posterior) and/or groin pain for >6 months; (2) reported hip/groin pain of ≥3 and <8 on a 11-point numerical pain rating scale with sport-specific movements (squatting, kicking, or cutting/change of direction); (3) had pain elicited in the hip/groin with the FADIR test; and (4) Kellgren-Lawrence (KL) grade <2 on hip radiographs.^18^ Each of the symptomatic participants’ hips were classified as either symptomatic or “other.” In symptomatic players, the contralateral hip was classified as “other” if the participant reported no hip/groin pain in that hip or had hip/groin pain but a negative FADIR test or had a KL grade ≥2. All hips in the control participants were asymptomatic and had a negative FADIR test in both hips.

Demographic data (including age, sex, body mass, and height) for both groups were collected at baseline and *t* tests were used to compare these data between the symptomatic and control groups.

### ROM Measures

**Figure 1.**
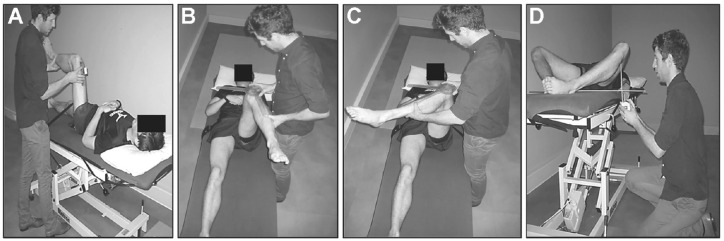
Methods of assessing hip range of motion: (A) flexion, (B) internal rotation, (C) external rotation, and (D) bent-knee fall out. (Adapted with permission from Crossley et al,^5^ CC BY-NC-ND 4.0.)

Hip joint ROM was assessed using 5 measures, with the methods of measurement described in detail in the supplementary files of the protocol publication^5^ and illustrated in Figure 1. Briefly, active flexion ROM was measured with a digital inclinometer placed on the thigh while the participant bent their knee toward their ipsilateral shoulder while the contralateral leg was stabilized to the plinth using a belt (Figure 1A). Passive internal and external rotation ROM were measured using a goniometer with the participant supine and their hip and knee flexed to 90°. The pelvis was stabilized using a belt (aligned to the anterior superior iliac spine), and the stationary arm of the goniometer was aligned with the pelvis, while the movement arm was aligned with the tibia (Figure 1, B and C). Total rotation ROM for each hip was calculated by summing internal and external rotation ROM. BKFO was assessed using established methods.^25,28^ The participant was positioned supine with their knees bent to 90° flexion. With the soles of their feet placed together, the participant allowed their knees to drop out toward the bed, and after gentle overpressure, the distance between the fibular head and the inferior aspect of a 1-m ruler placed on the bed was determined using a rigid tape measure (Figure 1D). The higher the measure of BKFO, the lower the hip ROM for this measure.

The interrater reliability was deemed excellent^23^ for all ROM measurement methods, with intraclass correlation coefficients (ICCs) ranging from 0.91 to 0.99 (Appendix Table A2).

### Radiographs

Each participant underwent a supine anteroposterior (AP) pelvis and a 45° Dunn radiograph of each hip. All radiographs were taken using standardized protocols at each radiology site. Bony hip morphology was determined using previously established semiquantitative methods.^1,2^ A point set was placed on predetermined locations on the surface of the femur and acetabulum using statistical shape modeling software (ASM toolkit, Manchester University). The alpha angle was then calculated using MATLAB v 7.1.0 (MathWorks).

Intraobserver reliability for cam morphology was measured by a single investigator (J.J.H.) completing 20 images twice, 1 week apart, and was determined to be good with respect to alpha angle on both AP radiograph (ICC = 0.92; 95% CI, 0.79-0.97) and 45° Dunn radiograph (ICC = 0.93; 95% CI, 0.84-0.97). Interobserver reliability ICCs were 0.76 for AP radiographs and 0.93 for 45° Dunn radiographs.

### Cam Morphology

Cam morphology was quantified on the AP pelvis and 45° Dunn views by the alpha angle as previously described in detail.^11,13^ Based on previously proposed threshold values,^3,38^ the presence of cam morphology was defined as an alpha angle ≥60° on either the AP or 45° Dunn views.

### Statistical Analysis

Data analyses were performed using Stata/IC 15.0 (StataCorp). All analyses were undertaken at a per-hip level. Linear regression models with generalized estimating equation (GEE) were used to examine the relationship between hip/groin pain presence/absence and hip ROM. The GEE analysis allowed for within-person correlation between a participant's hips. The presence of hip/groin pain was the independent variable, and each measure of hip ROM the dependent variable. Interaction terms of group (symptomatic or control) × cam morphology and group × sex were entered into each regression model. If an interaction were present (interaction term, *P* < .05), data were stratified by either cam morphology or sex as appropriate and explored in separate linear models controlling for age. If no interactions were present, the interaction terms were dropped, and the relationship between group and ROM was analyzed within the model, adjusting for cam morphology, sex, and age. The potential confounders (cam morphology, sex, and age) were selected based on evidence of clinical relevance and existing literature.^14,19,20,28,29,31^

A power calculation determined that 42 was the minimum number of participants required to detect a difference in ROM greater than the measurement error for each ROM measure with 90% power and an alpha level of 0.05.

## Results

### Participant Characteristics

Demographic data and cam morphology prevalence for the study participants are summarized in Table 1, with more detailed sex-specific demographics included in Appendix Table A3. No differences in demographic variables were observed between the participants with hip/groin pain and those without (Table 1). In 70 of the 184 players with hip/groin pain, the contralateral hip was excluded from all analysis as it was classified as “other” and did not fulfill study-inclusion criteria. Twelve participants (22 hips with hip/groin pain and 2 control hips) had AP but not 45° Dunn radiographs due to protocol deviations; these hips were excluded from the analyses. A standing and not a supine AP pelvis radiograph was taken in 7 participants (14 hips) with hip/groin pain, with these hips included in the overall analysis. Therefore, 184 participants (276 hips) with hip/groin pain and 50 controls (98 hips) were included in the analyses.

**Table 1:** Participant Characteristics^*a*^

Variable	Symptomatic (n = 184)	Control (n = 50)	*P*
Age, y	26 [24-30] (range, 18-49)	26 [23-31] (range, 20-46)	.757
Height, cm	178 ± 8.8 (range, 150-201)	178 ± 9.9 (range, 158-200)	.221
Body mass, kg	78.5 ± 12.8 (range, 46-121)	76.7 ± 13.5 (range, 51-104)	.396
BMI, kg/m^2^	24.7 ± 3.2 (range, 18.7-35.1)	24.3 ± 3.1 (range, 17.7-32.9)	.994
Sex, female	39 (21)	14 (28)	.268
Soccer players,^*b*^ n (%)	93 (51)	30 (60)	.235
Cam morphology present, number of hips/total hips (%)	195/276 (71)	60/98 (61)	.085
Alpha angle,^*c*^ deg	71 ± 15.3 (range, 38-112)	68.5 ± 15.4 (range, 43-104)	.112

a Data are presented as median [interquartile range] or mean ± standard deviation unless otherwise indicated. BMI, body mass index.

b The remaining participants played Australian Rules football.

c Largest of the alpha angles determined from the anteroposterior and Dunn view radiographs.

### Relationship Between Hip/Groin Pain and ROM

**Figure 2.**
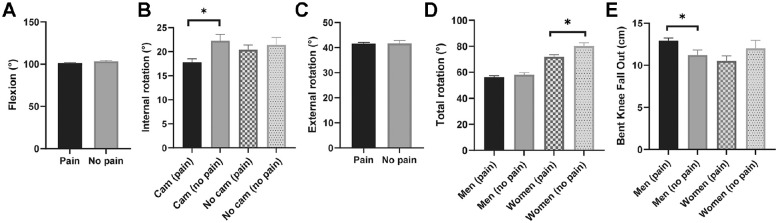
Results of the adjusted analyses examining the relationship between hip/groin pain and range of motion (adjusted mean and standard deviation): (A) flexion by group, (B) internal rotation stratified by cam morphology, (C) external rotation by group, (D) total rotation stratified by sex, and (E) bent-knee fall out stratified by sex. *Statistically significant on pairwise comparisons (*P* < .05).

The results of the analyses exploring the relationship between hip/groin pain and hip ROM are presented in Table 2 and Figure 2. Regarding internal rotation ROM, an interaction was present between hip/groin pain and cam morphology; symptomatic hips with cam morphology had less internal rotation ROM than control hips with cam morphology (adjusted mean difference = −4.5°; 95% CI, −7.4° to −1.6°; *P* = .029). Conversely, internal rotation ROM did not differ between symptomatic and control hips without cam morphology (adjusted mean difference = −1.0°, 95% CI, −4.6 to 2.6°; *P* = .579). For total rotation ROM, an interaction was present between hip/groin pain and sex. Women with hip/groin pain had lower total rotation ROM than women without hip/groin pain (adjusted mean difference = −8.2°, 95% CI, −14.1° to −2.2°, *P* = .036). Total rotation ROM did not differ between men with and without hip/groin pain (adjusted mean difference = −1.8°, 95% CI, −5.4° to 1.8°; *P* = .322). For BKFO, an interaction was found between hip/groin pain and sex. Men with hip/groin pain had lower BKFO ROM (ie, higher BKFO values) than men without (adjusted mean difference = 1.6 cm, 95% CI, 0.3 to 3.0 cm, *P* = .022). However, BKFO range did not differ between women with and without hip/groin pain (adjusted mean difference = −1.5 cm, 95% CI, −3.73 to 0.8 cm, *P* = .176). Flexion and external rotation ROM did not differ between the players with and without hip/groin pain.

**Table 2:** Results of the Adjusted Analyses Exploring the Relationship Between Hip/Groin Pain and Hip ROM^*a*^

ROM Measure	Adjusted Mean (95% CI)	*P* (Group)	Sex, Adjusted Mean (95% CI)		*P* (Group × Sex)	Cam Morphology, Adjusted Mean (95% CI)		*P* (Group × Cam)
			Male	Female		Cam	No Cam	
Flexion, deg		.078			.573			.235
Pain	101.2 (100.0-102.3)		—	—		—	—	
No pain	103.3 (101.2-105.5)		—	—		—	—	
Internal rotation, deg		—			.086			**.029**
Pain	—		—	—		17.8 (16.4-19.2)	20.4 (18.4-22.3)	
No pain	—		—	—		22.3 (19.8-24.9)	21.4 (18.3-24.4)	
External rotation, deg		.865			.419			.498
Pain	41.7 (39.4-43.9)		—	—		—	—	
No pain	41.4 (40.2-42.7)		—	—		—	—	
Total rotation, deg		—			**.036**			.244
Pain	—		56.4 (54.7-58.1)	71.9 (68.7-75.2)		—	—	
No pain	—		58.2 (55.1-61.4)	80.1 (75.0-85.2)		—	—	
BKFO, cm		—			**.022**			.857
Pain	—		12.9 (12.3-13.6)	10.5 (9.3-11.7)		—	—	
No pain	—		11.3 (10.1-12.5)	12.0 (10.1-13.9)		—	—	

a Dashes indicate areas not applicable. Boldface *P* values indicate statistically significant difference between groups (*P* < .05). BKFO, bent-knee fall out; ROM, range of motion.

## Discussion

We investigated the relationship between the presence of hip/groin pain and hip ROM in amateur soccer and Australian Rules football players. Hips with hip/groin pain and cam morphology (ie, hips fitting the definition of FAI syndrome^10^) were found to have lower internal rotation ROM than asymptomatic hips with cam morphology. Total rotation ROM was less in women with hip/groin pain but not men. Men with hip/groin pain had less BKFO range compared with controls, but in women, there was no relationship between hip/groin pain and BKFO range. No relationships were observed between hip/groin pain and flexion or external rotation ROM.

### Hip/Groin Pain and ROM: Accounting for Confounding Variables

The association between hip/groin pain and ROM has been synthesized in several systematic reviews^6,8,22,27,30,35,41^ and recent consensus statements.^15,29^ Conflicting evidence for an association created uncertainty in the recommendations of expert clinical researchers regarding the utility (or not) of examining ROM in these patients.^29^ Heterogeneity in diagnostic categorization of participants (including the presence of cam morphology) and measurement methods in included studies has contributed to this conflicting evidence.^15,29^ Cam morphology has been associated with lower hip internal and total rotation ROM, independent of symptoms.^26,29,35^ We found that internal rotation ROM was reduced only in the hips with pain and cam morphology (ie, hips that fit the definition of FAI syndrome as agreed upon in two international consensus meetings^10,33^). The mean difference between hips with cam morphology that were symptomatic compared with those which were asymptomatic was 4.5° which is greater than the measurement error for this method (2°).^29^ Furthermore, this ROM difference represents approximately 20% of the total measure, so we can be confident that this difference is clinically important. In our cohort, players with hip/groin pain but without cam morphology did not have reduced internal rotation ROM compared to control hips. This suggests that it is the combination of the presence of pain and cam morphology that was responsible for the reduced internal rotation ROM rather than either of these features in isolation. This finding may also explain why previous studies that did not examine for the presence of cam morphology in their cohort have not found an association between hip/groin pain and internal rotation ROM.

Total rotation ROM was less in women's hips with pain compared with asymptomatic hips (but not men’s), and BKFO was reduced in men's hips with pain (but not women’s). We can only speculate as to why these sex-related differences in the relationship between group and ROM were found, as it has not been examined previously. Differences between men and women in pelvic or femoral shape, neuromuscular recruitment patterns, adductor pain/increased tone, or biomechanics may explain these differences and should be explored in future research. Our findings may also explain conflicting evidence for an association between hip/groin pain and ROM given that previous cohorts have not used clear definitions for FAI syndrome, and not always taken into account sex and/or cam morphology presence.^6,8,35^ Our findings suggest that future research needs to adjust for cam morphology, sex, and age when examining ROM in participants with hip/groin pain.

### How Useful is Measuring ROM in Clinic Practice?

The clinical utility of measuring ROM may vary depending on the patient's sex and/or whether they have cam morphology. In our cohort, internal rotation ROM was lower in the presence of hip/groin pain and cam morphology, inferring that this ROM measure may be clinically useful in conjunction with imaging findings and symptoms when diagnosing patients with FAI syndrome.^10^ The adjusted mean difference between hips with cam morphology and symptoms and those without symptoms was 4.5°, greater than the standard error of measurement of 2° determined in this cohort and 4.3° in the study by Mosler et al^28^ using the same methods. While these small differences in internal rotation ROM may assist in diagnosis, the clinical usefulness of examining this measure as an impairment in these patients is currently unknown. Intervention studies that aim to improve internal rotation ROM and then examine its relationship with hip-related quality of life or pain might help answer this question. Total rotation ROM was lower in women with hip/groin pain (adjusted mean difference = −8.2°) but not in men, and BKFO ROM was lower in men (adjusted mean difference = −1.5 cm) but not in women. These mean differences were larger than the standard error of measurement for these measures (5.02° and 0.54 cm, respectively), indicating that they may be important to include in the assessment of a patient with hip/groin pain, depending on the patient's sex.

### Clinical Implications of the Study Findings

Our findings suggest that in amateur soccer and Australian Rules football players with hip/groin pain, ROM is a useful measure to include in clinical assessment, particularly internal rotation, total rotation, and BKFO. Clinicians should be aware that observed ROM impairments may vary between sexes and in the presence or absence of cam morphology and therefore should be considered when determining if ROM findings represent a clinically important impairment. Lower internal rotation ROM in patients with hip/groin pain and cam morphology (ie, FAI syndrome) may represent a treatment target due to the association with the development of end-stage hip osteoarthritis.^1-3^ Women with pain who present with less total rotation ROM, and men with pain and reduced BKFO may represent clinically relevant impairments to assess and treat. Uncertainty exists regarding the extent of ROM impairment that may impact a patient's presentation, or what ROM impairments mean for patients living with their condition over time. Following these participants longitudinally and determining if these ROM impairments have any effect on their future hip-related quality of life will allow us to better understand the potential relevance of these ROM impairments.

### Limitations

Our cohort included players with self-reported hip/groin pain and a positive FADIR test. Therefore, the pathoanatomic source of symptoms is not specifically known, and it is likely that extra-articular sources of pain, as outlined in the Doha agreement,^40^ were present in our cohort. The poor specificity of the FADIR test and our inability to specifically identify pain source in the hip means that our findings cannot be assumed to be specific to hip-related pain.^33^ Furthermore, with only 20% of our cohort being women, we may have been underpowered to detect further sex-related interactions in the relationship between hip/groin and ROM. In addition, our study participants were all still participating in their chosen sport, despite symptoms, so these findings may not apply to more severe pain presentations or other athletic or nonathletic populations.

We used the definition of cam morphology most commonly found in the literature,^38^ However, more sensitive measures of examining size and/or location of cam morphology may reveal associations between with hip/groin pain and ROM. Other bony morphological variables such as femoral torsion, acetabular shape, and version may be associated with ROM but were not examined in our study. Furthermore, other measures of ROM such as abduction or sport-specific combined measures were not included in our study for pragmatic reasons related to assessment time burden. The association between other ROM measures and hip/groin pain could be examined in future research to build the evidence of the clinical utility of measuring ROM in young, active adults with hip/groin pain.

## Conclusion

The key points of this study were as follows:

- **Findings:** Hip/groin pain is associated with reduced internal rotation ROM in young active people with cam morphology, but not when cam morphology is absent. Total rotation ROM is reduced in women but not men with hip/groin pain. BKFO range is reduced in men with hip/groin pain but not women.
- **Implications:** Our findings suggest that ROM is a useful measure to include in clinical assessment, as it is reduced in hips with hip/groin pain. However, clinicians need to consider the influence of cam morphology and sex on ROM measures in their clinical reasoning when determining the importance of the ROM measures assessed. Researchers should include the confounding variables of cam morphology, sex, and age in future analyses of hip joint ROM.
- **Caution:** Our findings are limited to the population studied, the ROM tests, and the bony hip morphological definitions used in our study. Of particular note, our study participants were still participating in their chosen sport, despite symptoms, so these findings may not apply to more severe pain presentations.

## Appendix

**Table A1:** Inclusion and Exclusion Criteria for Participants in the FORCe Cohort^*a*^

Symptomatic Group	Control Group
Inclusion Criteria	
• Age 18-50 years • Playing in subelite competition • Undertaking ≥2 sessions (games or training) of soccer or Australian Rules football per week • Self-reported hip (anterior/lateral/posterior) and/or groin pain fulfilling criteria: (1) gradual onset; (2) duration >6 months; (3) average hip/groin pain of >3 and <8 on 11-point NPRS^*b*^ with sport-specific movements. • Positive FADIR test in at least 1 hip	• Age 18-50 years • Playing in subelite competition • Undertaking ≥2 sessions (games or training) of soccer or Australian Rules football per week • Negative FADIR test in both hips
Exclusion Criteria	
• Self-reported history of a significant hip and/or groin condition, specifically: bursitis, congenital dislocation of the hip, fracture, osteochondritis dissecans, Legg-Calvé-Perthes disease, septic or rheumatoid arthritis, slipped capital femoral epiphysis, or subluxations/dislocations • Previous hip and/or pelvis surgery • KL grade of ≥2 on AP pelvis radiograph • Any lumbar spine or lower-limb injury/complaint in the previous 3 months (eg, hamstring muscle injury or sprained ankle) that resulted in the inability to fully bear weight or undertake testing procedures • Contraindications to radiographs (eg, pregnancy) • Received intra-articular hip injection (of any type) in the previous 3 months • Unable to understand spoken and written English	• Self-reported history of hip and/or groin pain or a significant hip and/or groin condition (as specified in symptomatic group exclusion criteria) • Lower-limb surgery (eg, ACL reconstruction) • KL grade of ≥2 on AP pelvis radiograph • Any lumbar spine or lower-limb injury/complaint in the previous 3 months (eg, hamstring muscle injury or sprained ankle) that resulted in the inability to fully bear weight or undertake testing procedures • Contraindications to radiographs (eg, pregnancy) • Unable to understand spoken and written English

a ACL, anterior cruciate ligament; AP, anteroposterior; FADIR, flexion, adduction, and internal rotation; KL, Kellgren-Lawrence; NRPS, numeric pain rating scale.

b Use of the NRPS in symptomatic players was a deviation from the original study protocol.

**Table A2:** Interrater Reliability for ROM Measures^*a*^

Test	Test 1, mean ± SD	Test 2, mean ± SD	ICC (95% CI)	SEM	SEM %	MDC
Flexion ROM, deg						
Dominant	109.35 ± 10.19	107.63 ± 11.43	0.95 (0.86-0.98)	2.28	2.1	6.31
Nondominant	111.20 ± 10.12	110.22 ± 11.91	0.93 (0.81-0.98)	2.68	2.4	5.25
BKFO, cm						
Dominant	9.52 ± 4.53	9.33 ± 4.15	0.99 (0.97-1.00)	0.45	4.8	1.26
Nondominant	9.60 ± 3.64	9.17 ± 3.40	0.97 (0.90-0.99)	0.63	6.7	1.75
ER ROM at 90° flexion, deg						
Dominant	41.40 ± 12.10	43.57 ± 13.62	0.96 (0.88-0.99)	2.42	5.7	6.70
Nondominant	44.57 ± 13.00	44.80 ± 15.35	0.95 (0.87-0.98)	2.90	6.5	8.05
IR ROM at 90° flexion, deg						
Dominant	21.73 ± 6.25	21.40 ± 6.59	0.91 (0.76-0.97)	1.88	8.7	5.20
Nondominant	23.70 ± 10.02	23.20 ± 8.51	0.95 (0.86-0.98)	2.24	9.6	6.21

a BKFO, bent-knee fall out; CI, confidence interval; ER, external rotation; ICC, intraclass correlation coefficient (2,1); IR, internal rotation; MDC, minimal detectable change; ROM, range of motion; SEM, standard error of measurement.

**Table A3:** Participant Characteristics Stratified by Sex^*a*^

	Symptomatic		Control	
	Men (n = 146)	Women (n = 38)	Men (n = 36)	Women (n = 14)
Age, y	27 [24-31] (range, 18-48)	26 [23-30] (range, 21-49)	27 [24-32] (range, 21-46)	26 [22-29] (range, 20-32)
Height, cm	181 ± 6.6 (range, 161-201)	167 ± 7.4 (range, 150-188)	181 ± 8.2 (range, 162-200)	168 ± 6.9 (range, 158-185)
Body mass, kg	82.0 ± 10.6 (range, 63-121)	65.6 ± 11.9 (range, 46-97)	81.2 ± 11.9 (range, 58-104)	65.4 ± 10.5 (range, 51-84)
BMI, kg/m^2^	25.0 ± 3.1 (range, 20-35)	23.3 ± 3.3 (range, 19-32)	24.7 ± 2.9 (range, 19-33)	23.1 ± 3.4 (range, 18-31)
Soccer players,^*b*^ n (%)	71 (49)	22 (58)	26 (72)	4 (29)
Cam morphology present, number of hips/total hips (%)	170/218 (78)	25/58 (43)	53/70 (76)	7/28 (25)

a Data are presented as median [interquartile range] or mean ± SD unless otherwise indicated. BMI, body mass index.

b The remaining participants played Australian Rules football.
